# Evolving insights into the improvement of adoptive T-cell immunotherapy through PD-1/PD-L1 blockade in the clinical spectrum of lung cancer

**DOI:** 10.1186/s12943-023-01926-4

**Published:** 2024-04-24

**Authors:** Yutao Li, Amit Sharma, Ingo G.H. Schmidt-Wolf

**Affiliations:** 1 Department of Integrated Oncology, Center for Integrated Oncology (CIO) Bonn, University Hospital Bonn, Venusberg Campus 1, D-53127, Bonn, Germany; 2 Department of Neurosurgery, University Hospital Bonn, Bonn, Germany

**Keywords:** Cytokine-induced killer cells, Chimeric antigen receptor T-cells, γδ T cells, Immune checkpoint

## Abstract

Undeniably, cancer immunotherapies have expanded the spectrum of cancer treatment, however, some patients do not respond to immunotherapies. This scenario is no different for lung cancer, whose two main types, non-small cell lung cancer (NSCLC) and small cell lung cancer (SCLC), still pose a serious clinical challenge. Adoptive T-cell therapies (ATC), which primarily include cytokine-induced killer (CIK) cell therapy, chimeric antigen receptor T-cell (CAR T-cell) therapy and γδ-T-cell therapy, strengthen the patient’s immune system in combating cancer. Combining ATC with immune checkpoint inhibitors (ICI) further enhances the effectiveness of this approach to eradicate cancer. With a particular emphasis on CIK cell therapy, which recently completed 30 years, we highlight the role of the PD-1/PD-L1 axis in NSCLC and SCLC. Besides, we provide insights into the potential synergies of PD-1/PD-L1 inhibitors with adoptive T-cell immunotherapy in reshaping the treatment paradigm for lung cancer.

## Introduction

Lung cancer remains the second most commonly diagnosed cancer worldwide [1], being categorized into two main types: non-small cell lung cancer (NSCLC) and small cell lung cancer (SCLC). Approximately 85% of patients exhibit a group of histological subtypes referred as NSCLC, of which lung adenocarcinoma (LUAD) and lung squamous cell carcinoma (LUSC) represent the most common subtypes [2]. In the United States, 5-year survival between 2008 and 2014 was 24% for all patients with NSCLC and 5.5% for those with distant metastases [3]. Likewise, the median overall survival of German advanced NSCLC patients was equally as low as that of other countries as shown in a German retrospective data analysis [4]. Unlike NSCLC, the SCLC accounts for approximately 13%-15% of all lung cancers with a 5-year survival rate of less than 7%, a rapid doubling time and a high propensity to metastasize. SCLC is considered a “recalcitrant” cancer as no significant improvements in survival and therapeutic approaches have been achieved for more than 30 years [5, 6].

Since cancer cells have several mechanisms to evade immune surveillance, including the PD-1/PD-L1 axis, this axis has also been focused on for LC. Thus, the programmed cell death protein 1 (PD-1 [also known as CD279]) and its ligand, programmed death ligand 1 (PD-L1 [also known as CD274]), have been utilized in several standard first-line LC treatments. PD-L1, also known as Cluster of Differentiation (CD274) or B7 homolog 1 (B7-H1), belongs to the growing B7 family of immune molecules and is involved in the regulation of cellular and humoral immune responses. B7-H1 belongs to the cell surface immunoglobulin superfamily with two Ig-like domains in the extracellular region and a short cytoplasmic domain within the extracellular region and a short cytoplasmic domain. Subsequent to nivolumab [7, 8], there have been additional PD-1 (pembrolizumab and cemiplimab) and PD-L1 (atezolizumab and durvalumab) inhibitors approved by the FDA. Unlike NSCLC cells, the efficacy of a combination of nivolumab and ipilimumab was enhanced in patients with high tumor mutation burden (TMB) in the nonrandomized or randomized cohorts of CheckMate 032 [9], possibly due to the nearly universal association of SCLC with smoking [10, 11]. However, in addition to improving survival, PD-1/PD-L1 inhibitors were associated with a wide range of unfavourable effects, for instance, immune-related adverse events (irAEs), including rash, colitis, hepatitis, endocrinopathies, and pneumonitis [12]. Thus, it is imperative to explore new therapeutic strategies to alleviate irAEs and improve the efficacy of immune effector cells.

Undeniably, the availability of the PD-1/PD-L1 axis also opens up the avenue for its combination with various cancer immunotherapies. One among them is cytokine-induced killer (CIK) cell therapy, pioneered by Ingo Schmidt-Wolf, et al. in 1991 [13]. CIK cells were described to have dual cytotoxic functions of innate immunity via NK-specific activating receptors and adaptive immunity via polyclonal TCR repertoire [14], and more than 80 clinical trials involving CIK cells ranging from solid tumors to hematologic malignancies (clinicaltrial.gov). Concerning CIK treatment for lung cancer, 12 clinical trials have been conducted. Of these, 10 studies reported that CIK treatment improved median progression-free survival, and 7 studies improved overall survival [15]. Like CIK cells, another alternatively novel immunotherapy chimeric antigen receptor T-cell (CAR-T) also showed great promise for NSCLC. However, unlike hematologic malignancies, the clinical application of CAR-T cells has remained limited success due to on-target/off-tumor as well as neurological toxicity [16]. Meanwhile, γδ T cell immune therapy also presented promising outcomes in a recent clinical trial on lung cancer patients [17]. Nevertheless, it is reasonable to optimize CIK therapy, for instance, a combination of PD-1/PD-L1 inhibitor based on T cell immune therapy. Particularly, the response of cancer cells to immunotherapy will be determined by both intrinsic properties of the cancer cells and specific interactions with the microenvironment [18]. In this review, we will discuss the PD-1/PD-L1 axis in the lung cancer microenvironment, current T cell adoptive immunotherapy combined with PD-1/PD-L1 blockade combination, as well as future directions.

## Landscape of PD-1/PD-L1 axis in NSCLC and SCLC

### The role of PD-1/PD-L1 in the tumor immune microenvironment of NSCLC

#### Cancer stem cell (CSC)

**Fig. 1:**
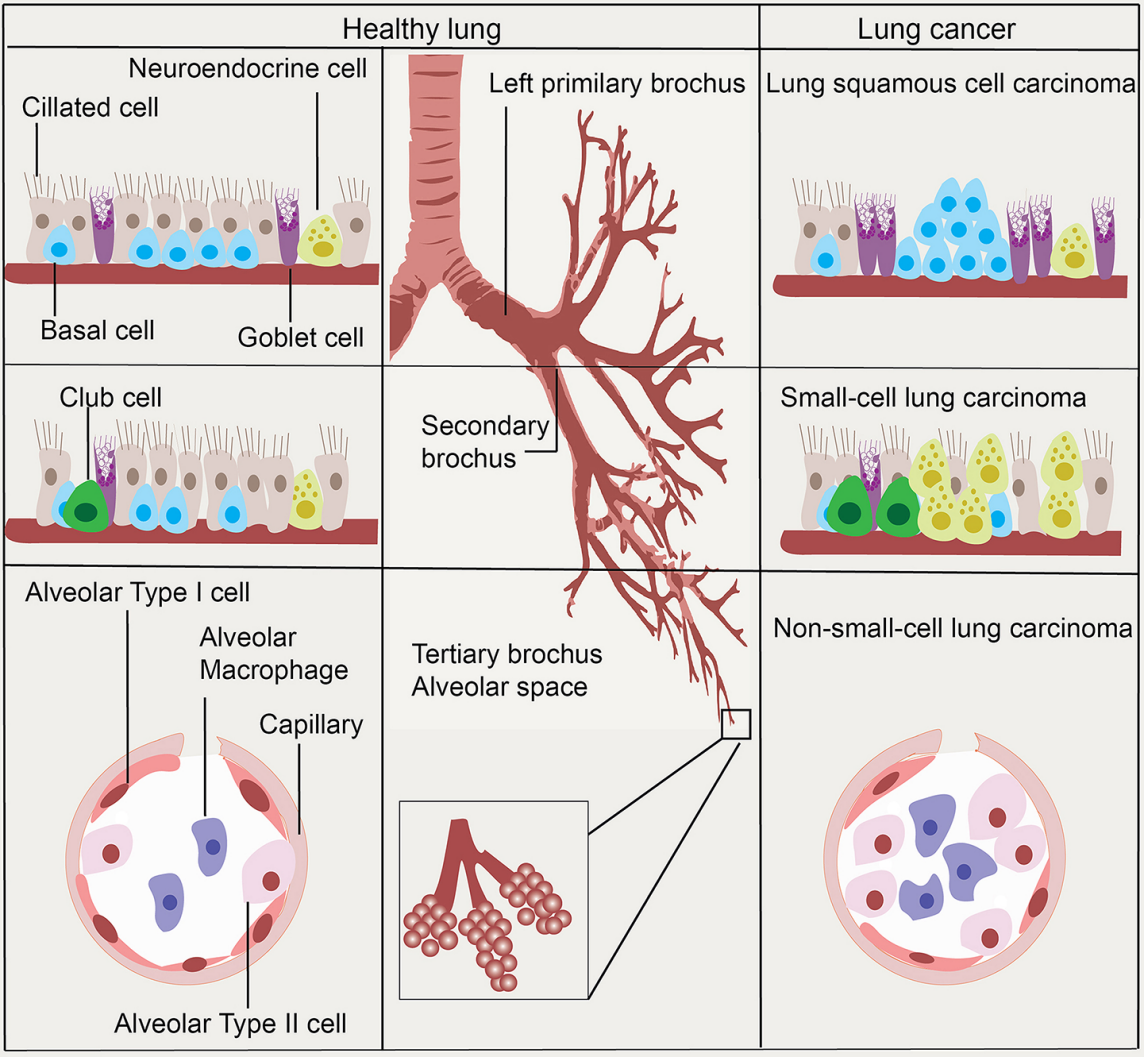
The putative origin of lung cancer stem cells in lung anatomical sites. In the trachea and main bronchi, airway basal cells are considered as a stem cell population, precursors of squamous cell lung carcinoma. Pulmonary neuroendocrine (PNE) cells are thought to serve as a precursor for the progress of small cell lung carcinoma. It is also believed that adenocarcinomas can originate from broncho alveolar stem cells or pneumocytes of type I and type II

Human lungs are composed of two distinct areas: the conducting airway, including the trachea, bronchi, and bronchioles, and the gas exchange regions, alveolar spaces. The division of these stem cells is thought to be sufficient to renew the lung’s structure of the lung during normal adult life. In the trachea and main bronchi, the tracheal epithelium consists mainly of columnar and mucus secreting goblet cells. In addition, airway basal cells are considered as a stem cell population, which can maintain the balance between their proliferation and differentiation. The imbalance can contribute to squamous cell metaplasia or dysplasia, which are precursors of squamous cell lung carcinoma [19, 20]. Meanwhile, in bronchioles and alveoli, non-ciliated club cells (Clara cells) are located in the bronchiolar and alveolar epithelium and could differentiate into ciliated cells after exposure to oxidant induced damage [21, 22]. Club cells are shown to survive KRAS mutations and to form lung tumors after tobacco carcinogen exposure [23]. The high frequency of club cell-like cells in papillary adenocarcinoma could be a useful histological marker for ALK^+^ lung cancers [24]. In addition, Club and Alveolar type 2 (AT2) cells give rise to EML4-ALK lung adenocarcinoma in mice model [25]. Pulmonary neuroendocrine (PNE) cells are thought to serve as a precursor for the progress of small cell lung carcinoma. Clara cells are not necessary for PNE cell hyperplasia since proliferation and hyperplasia of PNE cells occurred in the conditional Clara cell ablation [25]. It is also believed that adenocarcinomas can originate from broncho alveolar stem cells or pneumocytes of type I and type II [26]. (Fig. 1).

**Fig. 2:**
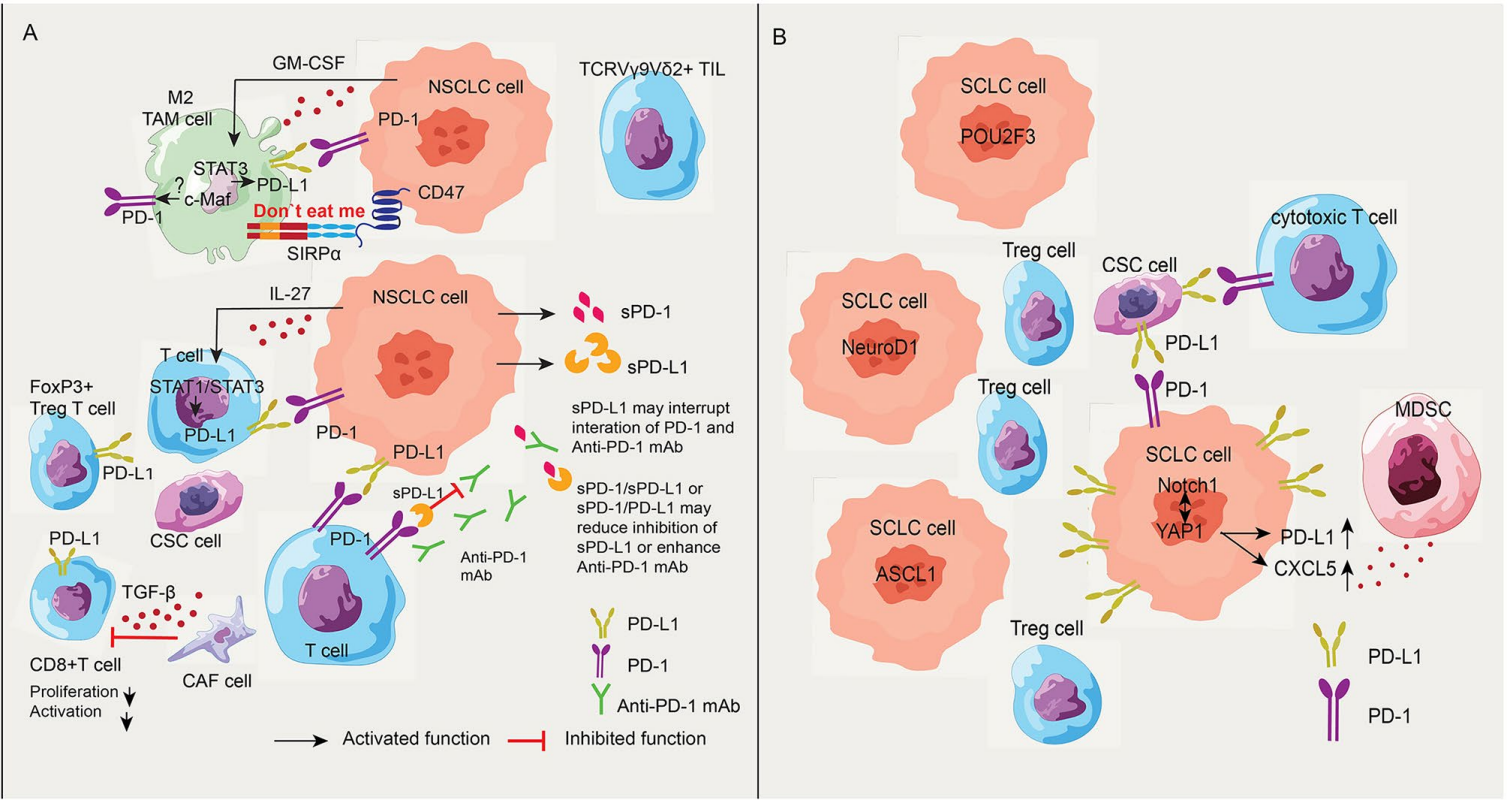
Schematic representation of the negative regulation of anti-tumor immune responses of PD-1/PD-L1 in NSCLC and SCLC. (**A**) PD-L1 expression in lung cancer cell lines was significantly upregulated by co-culture with M2-differentiated tumor-associated macrophages (TAMs). PD-L1 overexpression on macrophages might be induced via STAT3 activation by cancer cell-derived GM-CSF. The interaction of CD47 on tumor cells and signal‐regulated protein (SIRPα) expressed on the surface of macrophages can protect cells from being “eaten” by macrophages in NSCLC patients. TGF-β secreted from cancer-associated fibroblasts (CAF cells) also reduced the proliferation and activation of CD8^+^ T cells. In addition, PD-L1^+^ lung cancer stem (CSC) cells as well as FoxP3^+^ Treg T cells may modify the metastatic lymph-node immune microenvironment in NSCLC patients. Besides, soluble PD-L1 (sPD-L1) might interrupt PD-1 and Anti-PD-1 mAb whereas interaction of sPD-1/sPD-L1 or sPD-1/PD-L1 may reduce inhibition of sPD-L1 or enhance Anti-PD-1 mAb. The interaction of PD-1/PD-L1 between TCRVγ9Vδ2^+^γδ tumor-infiltrating lymphocytes (TILs) and αβ T cells could restrain the activation of T cells. Intratumoral Vδ1 T cells demonstrated natural killer and CD8^+^ T cell function. (**B**) The role of PD-L1 in SCLC. In the SCLC-Y subtype, YAP1 not only affects PD-L1, but also upregulates CXCL5 to recruit myeloid-derived suppressor cells (MDSCs). YAP1 and Notch1 are complementary and each suppresses neuroendocrine (NE) differentiation

CSCs are thought to be responsible for cancer initiation, progression, metastasis, recurrence, and drug resistance. The presence of CSCs with PD-L1 expression in the metastatic lymph nodes (LNs) in lung cancer patients might correlate with immunotherapy [27]. Additional data showed that PD-L1^+^ lung cancer stem cells modified the metastatic lymph-node immune microenvironment in NSCLC patients, positively correlated with the percentage of Tregs, PD-1^+^ CD4^+^ T cells and Tim3^+^ CD4^+^ T cells, while negatively correlated with that of CD4^+^ T cells and CD28^+^ CD4^+^ T cells [28]. Indeed, this suppressive immunophenotype correlated with tumor PD-L1 can be evaluated by endobronchial ultrasound-guided transbronchial needle aspiration before immune therapy [29] (Fig. 2A).

#### Tumor-associated macrophages (TAMs)

Tumor-associated macrophages (TAMs) play an important role on the tumorigenesis of lung cancer. TAMs conduct pro-angiogenic effects and polarization of TAMs can affect the proliferation, migration, invasion. TGF-β and IL-10 secreted from TAMs are important factors that form the microenvironment of immunosuppressive tumors [30]. PD-L1 on cancer cells engages with PD-1 on immune cells, contributing to cancer immune escape [31]. Additionally, TAMs are known to be instrumental in the immunosuppressive effects of the PD-1/PD-L1 pathway in the cancer microenvironment. PD-L1 was significantly higher in macrophages in both the tumor and stromal compartment compared with other immune cells in NSCLC patients, correlating with better overall survival [32]. This self-protective immune escape could aid cells to evade being eliminated by neighboring cells, which might also benefit from PD-1/PD-L1 inhibitors. PD-L1 overexpression on macrophages induced via STAT3 activation by cancer cell-derived GM-CSF was suggested to promote cancer progression in lung adenocarcinoma in vitro and vivo animal models [33]. Furthermore, data from lung adenocarcinoma patients has confirmed that high PD-L1 expression on macrophages was correlated with the presence of EGFR mutation, a lower cancer grade, and a shorter cancer-specific overall survival [33]. Watanabe H. et al. reported a case of a 72-year-old man with PD-L1-nagative lung adenocarcinoma harboring an EGFR mutation who responded to nivolumab for more than 2 years. The pathological evidence demonstrated infiltration of PD-L1^+^ TAM and CD8^+^ lymphocytes in the tumor environment, revealing that PD-L1 high expression in TAM might be an indicator of a positive response to anti-PD-1 antibodies [34]. Another study revealed that in patients with early-stage lung adenocarcinoma, expression of PD-L1 on the cell surface of tumor cells was observed, which was accompanied by an increase in TAMs, cytotoxic CD8^+^ T cells, and regulatory FoxP3^+^ T cells [35]. Additional in vitro investigations revealed that PD-L1 expression in lung cancer cell lines was significantly upregulated by co-culture with M2-differentiated macrophages, whereas it was downregulated by a transforming growth factor‐β inhibitor. It is known that the interaction of CD47 on tumor cells and signal‐regulated protein (SIRPα) expressed on the surface of macrophages can protect cells from being “eaten” by macrophages, as has been reported in NSCLC patients. And that dual targeting of CD47/PD-L1 innate and adaptive checkpoints may serve as new combined dual-targeting immunotherapy to enhances macrophage phagocytosis [36]. Taken together, tumor‐infiltrating TAM are extrinsic regulators of tumor PD‐L1 expression, indicating that combination therapy targeting both tumor PD‐L1 and stromal TAM molecular might be a possible strategy for effective treatment of lung cancer (Fig. 2A). Recently, Carfilzomib modulates tumor microenvironment via driving M2 macrophages to express M1 cytokines to potentiate PD-1 antibody immune therapy for lung cancer [37]. Liu M. et al. suggested that the transcription factor c-Maf critically regulates human M2 macrophages/monocytes infiltrating the tumor and circulating monocytes from patients with NSCLC, as inhibition of c-Maf partially overcomes resistance to anti-PD-1 therapy in a subcutaneous LLC tumor model [38]. How c-Maf promotes immunoregulation of PD-1-expressed TAMs or CD8^+^ T cells in NSCLC patients is still unknown, while it has been already been demonstrated in multiple sclerosis and relapsed/refractory classic Hodgkin lymphoma [39, 40].

#### Cancer-associated fibroblasts (CAF)

Some clinical studies on immunotherapy via immune checkpoints emphasized the role of TGF-β. In metastatic urothelial cancer (mUC), TGF-β attenuates tumor response to PD-L1 blockade (atezolizumab) by contributing to exclusion of T cells [41]. The lack of response was associated with a transforming growth factor β (TGF-β) signature in fibroblasts, particularly in patients with CD8^+^ T cells excluded from the tumor parenchyma and instead found in the fibroblast and collagen-rich peritumoral stroma. In an animal model, these immune exclusion properties were also recapitulated, and a TGF-β-blocking antibody together with anti-PD-L1 reduced TGF-β signaling in stromal cells, facilitated T cell entry into the center of the tumor, and elicited potent anti-tumor immunity [42]. TGF-β signaling primarily mediates extracellular matrix (ECM) remodeling in the human NSCLC cell line A549. Ln-γ2, a member of the laminin family of ECM, was transcriptionally activated by TGF-β1 secreted from cancer-associated fibroblasts via JNK/AP1 signaling, and the mediated cell exclusion attenuates the response to anti-PD-1 therapy [42] (Fig. 2A).

#### TCR-αβ T cells exhaustion

T cell repertoire analysis has revealed that in early-stage NSCLC patients, there is greater homology of the T-cell repertoire between the tumor and the uninvolved tumor-adjacent lung, suggesting a less tumor-focused T-cell response as well as an association with inferior survival. Furthermore, the accumulation of regulatory CD4^+^ T cells in the tumor center may impair the ability of CD8^+^ T cells to proliferate in response to antigens [43], although the increased proliferation of PD-1^+^ CD8^+^ T cells in peripheral blood after PD-1-targeting therapy in lung cancer patients may overcome it [44]. Conversely, PD-L1^+^ CD8^+^ T cells exerted regulatory functions that inhibit CD8^+^ T cell proliferation and cytotoxic capabilities via the PD-L1/PD-1 axis. Moreover, tumor-derived IL-27 promotes PD-L1^+^ CD8^+^ T cell development through STAT1/STAT3 signaling [45]. Therefore, it is plausible that adoptive T-cell therapy combined with PD-1/PD-L1 inhibitors might increase clinical benefits because of cytotoxic T-cell infusion (Fig. 2A).

#### Soluble PD-1/PD-L1

Soluble PD-1/PD-L1 forms are generated by proteolytic shedding or alternative splicing of pre-mRNA, presenting an active circulating protein, with immune-modulatory functions [46]. In the recent study, PD-L1–vInt4, a splicing variant of PD-L1, was reported to be detectable in clinical samples of lung squamous cell carcinoma (LUSC) and its secretion resisted anti–PD-L1 antibody treatment via alternative polyadenylation. It is noteworthy that several LUSC samples presented a lack of capability of antigen presentation due to the loss of HLA expression overexpression in vitro experiment of PD-L1–vInt4 in vitro experiment [47]. Besides, sPD-L1expression in plasma of small cell lung cancer is associated with disease progression [48]. In contrast, some animal outcomes have been documented the anti-tumor effect of sPD-1 [49, 50]. An increased or stable sPD-1 level independently correlated with longer PFS in two cycles of nivolumab-treated metastatic NSCLC patients, suggesting sPD-1 as a predictive biomarker of response to ICI treatment in patients with lung cancer [51, 52]. Considering the analysis of sPD-L1 with immune-assays (ELISA) method may not distinguish between vesicular and soluble forms in circulation of cancer patients in some studies, dynamic blood PD-L1 expression for immune checkpoint inhibitors in advanced NSCLC patients might be more reliable as a biomarker [53]. Nevertheless, further work is required to better understand the functions of soluble PD-1/PD-L1 variants in lung cancer (Fig. 2A).

#### TCR-γδ T cells tumor-infiltrating lymphocytes (TILs)

γ/δ T lymphocytes localize in different epithelial tissues and are phenotypically distinct from peripheral γ/δ T cell-population. In about one-fourth of human lung cancers, γ/δ T cells represented a significant proportion of freshly isolated tumor-infiltrating lymphocytes [54]. Ferrarini M. et al. found that half of the Vδ1^+^ (as well as Vδ1^−^Vδ2^−^) γ/δ-lymphocytes that could be selectively expanded from human lung cancers also co-expressed the CD8α/α homodimer [55]. Furthermore, it has been reported that TCS1^+^ γ/δ^+^ tumor-infiltrating lymphocytes from human lung carcinomas lysed only autologous tumor cells and K-562 [56]. Human normal lung tissues and NSCLCs harbor resident populations of γδ T cells, particularly enriched in the Vδ1 subtype. Intratumoral Vδ1 T cells exhibited stemness characteristics and were skewed toward cytolysis and helper T cell type 1 function, similar to intratumoral natural killer and CD8^+^ T cells, which are considered beneficial to the patients [57]. Based on deconvolution of human cancers microarrays, TCRVγ9Vδ2^+^γδ TIL abundance in lung carcinoma was also reassessed by a study in ∼10,000 cancer biopsies. However, TCRVγ9Vδ2^+^ γδ TIL was not positively correlated with cytolytic activity and favorable outcome like αβ TIL [58]. In contrast, Cazzetta V. et al. revealed that NKG2A expression identifies a subset of human Vδ2 T cells exerting the highest antitumor effector functions [59]. This apparent discrepancy illustrates the complexity of the relationship between TCRVγ9Vδ2^+^ γδ TIL and lung cancer and also depends on differences in measurement methodologies (whole-tissue transcriptomic analysis, phenotypic analysis or immunohistochemistry analysis).

### The role of PD-1/PD-L1 in small-cell lung carcinoma (SCLC)

SCLC is broadly classed as limited stage-small cell lung cancer (LS-SCLC) and extensive stage- small cell lung cancer (ES-SCLC). Although SCLC harbors a high mutation rate (tumor mutational burden/TMB, a biomarker of sensitivity to immunotherapy in SCLC) [60], the pooled prevalence of PD-L1 expression stands at 26.0%, and 22.0% respectively, after excluding the potential outlier studies, which remains lower compared to NSCLC [61]. In addition, low expression of MHC-I, which leads to a decrease of cytotoxic T-lymphocytes (TILs) infiltrating the SCLC tumor, and excess of regulatory T cells (Tregs), which can inhibit activation, expansion and effector functions of other T cells [62], may contribute to low response to immune checkpoint inhibitors (ICIs) (approximately 10% with anti-PD-1 monotherapy) [63, 64].

In SCLC, small cell lung cancer stem cells display mesenchymal properties and exploit immune checkpoint pathways in activated cytotoxic T lymphocytes [65]. A subpopulation of pulmonary neuroendocrine cells are reserve stem cells regulated by the tumor suppressors Rb, p53, and Notch, and are considered tumor-initiating cells for SCLC [66].

A new classification of SCLC subtypes has been defined by differential expression of four key transcription regulators: achaete-scute homologue 1 (ASCL1; also known as ASH1), neurogenic differentiation factor 1 (NeuroD1), yes-associated protein 1 (YAP1) and POU class 2 homeobox 3 (POU2F3) [67]. YAP1 has been shown to contribute to inducing immunosuppressive TME by upregulating PD-L1 [68] or stimulating cytokines such as CXCL5 from tumor cells to recruit tumor-infiltrating macrophages, myeloid-derived suppressor cells (MDSCs) [69] and Tregs cells. In fact, it has been associated with mutations in the phosphatidylinositol 3-kinase (PI3K)/AKT/mTOR signaling pathway [70, 71]. Additionally, Notch signaling has been documented to play a critical role in the response to ICB in SCLC [72]. YAP1 and Notch1 are complementary and may be involved in cell proliferation, EMT, drug resistance, and neuroendocrine (NE) differentiation [73]. The mechanism of SCLC has not been fully understood (Fig. 2B).

### The possible mechanism of PD-1/PD-L1 in NSCLC cells

Several extrinsic factors (e.g., release of interferon-γ by immune cells that upregulate PD-L1 expression) and intrinsic factors regulate PD-L1 expression in NSCLC. As one of extrinsic factors, environmental tobacco smoke (ETS), contributes to a distinct PI3K (phosphatidylinositol 3-kinase)–Akt pathway that leads to cell survival in adenocarcinoma [74]. Genomic alterations that activate KRAS, EGFR, and ALK, as well as the loss of PTEN, have been associated with increased PD-L1 expression. Several tumorigenic intrinsic factors such as activation of the mechanistic target of rapamycin (mTOR), mitogen-activated protein kinase (MAPK) and Myc pathways can increase PD-L1. Alternatively, methylation, allelic loss and gene silencing expression might be considered [75]. Also, the molecular involvement of PD-L1 single nucleotide polymorphisms and non-synonymous single nucleotide polymorphisms (nsSNPs) with oncogenic potential in NSCLC, cannot be excluded [76, 77].

**Fig. 3:**
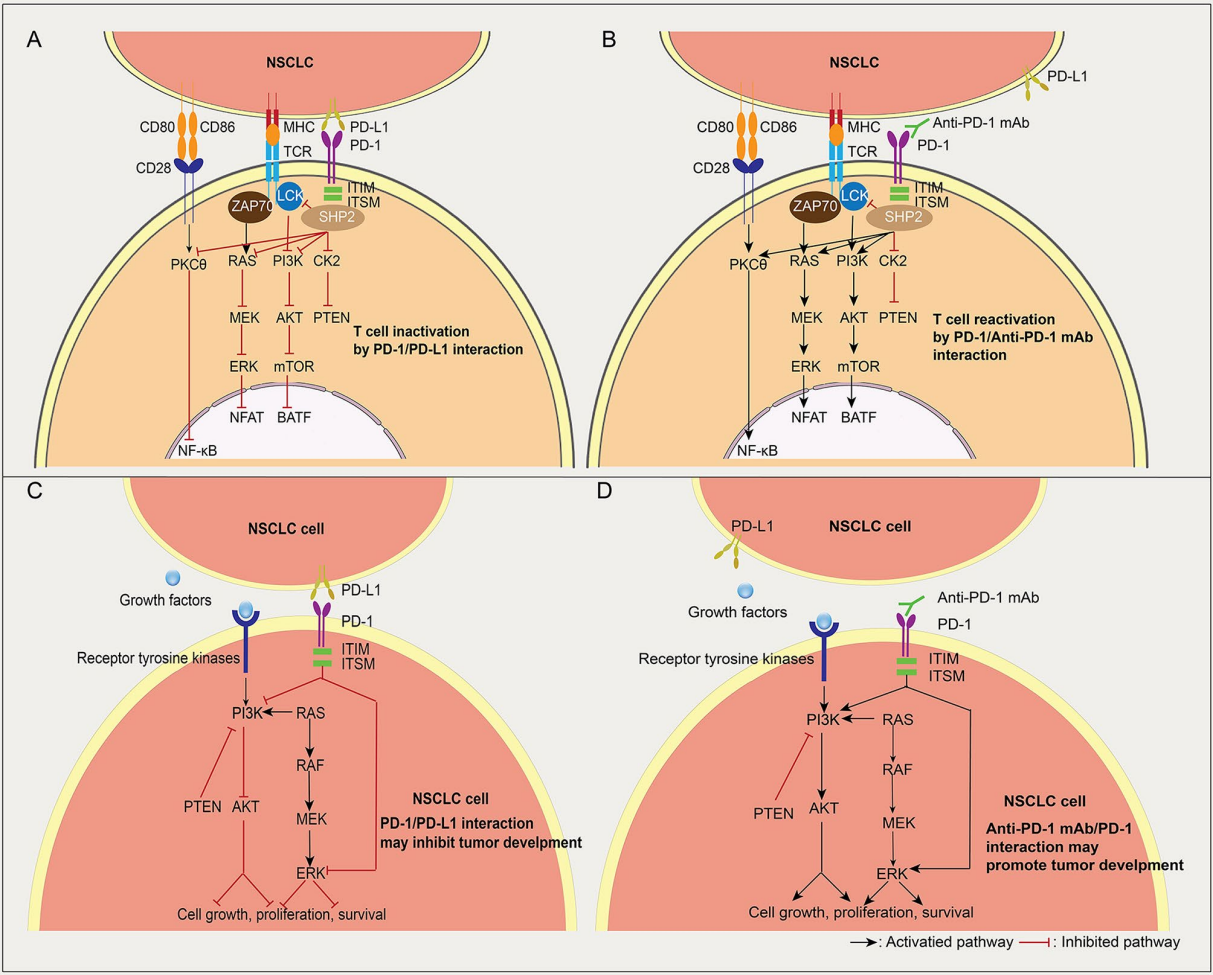
Interaction of PD-1/PD-L1 between T cells and NSCLC cells. (**A-B**) The engagement of PD-1 in T cells and PD-1 ligands leads to the recruitment of SHP-1/2 (Src homology 2-containing tyrosine phosphatase 1/2) to the C-terminal of the ITSM. SHP-2 then dephosphorylates TCR-associated CD-3ζ and ZAP70, resulting in the inhibition of downstream signaling and T cell inactivation (**A**). In the presence of Anti-PD-1 mAb, T cells might be reactivated via PD-1/PD-L1 axis (**B**). (**C-D**) The effect of tumor cell-intrinsic PD-1 on NSCLC cells. PD-L1 expressed by NSCLC cells or other cells acts on PD-1^+^ tumor cells to mediate PD-1 signaling in tumor cells via ITIM and ITSM. The AKT and ERK1/2 pathways can suppress tumor growth by dampening AKT and ERK signaling (**C**). Anti-PD 1 mAb blocks PD-1/PD-L1-mediated tumor suppression, leading to hyperprogression in NSCLC cells (**D**)

PD-1, is a type I transmembrane protein encoded by the PDCD1 gene of the CD28 immunoglobulin superfamily, comprised of a single Ig variable-type (IgV) extracellular domain, a transmembrane domain and a cytoplasmic domain. N-terminal and C-terminal tyrosine residues in the cytoplasmic domain are involved in the formation of immunoreceptor tyrosine-based inhibitory motifs (ITIMs) and immunoreceptor tyrosine-based switch motifs (ITSMs) [78]. The engagement of PD-1 in T cells and PD-1 ligands leads to the recruitment of SHP-1/2 (Src homology 2-containing tyrosine phosphatase 1/2) to the C-terminal of the ITSM. SHP-2 then dephosphorylates TCR-associated CD-3ζ and ZAP70, resulting in the inhibition of downstream signaling [79]. The advent of human T cell non-Hodgkin lymphomas and relative animal models has revealed that the activity of PD-1 enhances levels of the tumor suppressor PTEN and attenuates signaling by the kinases PI3K/AKT and PKCθ/NF-κB pathways in oncogenic T cells [80]. PD-1 on CD4^+^ T regulates cell cycle and inhibits T cell proliferation via Akt and Ras pathways [81]. Moreover, Lck signaling has been shown to contribute to PD-1^+^ T cell exhaustion via CD28 co-stimulation in a humanized mouse model [82] (Fig. 3A and B).

In recent years, the role of intrinsic PD-1 in NSCLC cells raises consideration. Wang X. et al. proposed that PD-1 and PD-L1 reduces tumor growth by suppressing AKT and ERK1/2. In the absence of an adaptive immune system, tumor cell-intrinsic PD-1/PD-L1 mediates the resistance to anti-PD-1/PD-L1 antibodies by activating AKT and ERK1/2, which induces tumor growth [83] (Fig. 3C and D).

## Current adaptive T cells clinical trials combined with PD-1/PD-L1 blockades in lung cancer

### Blockade of PD-1 immunosuppression boosts CIK/DC-CIK therapy

Shortly after injection of CIK cells, a bioluminescent signal was detected in the lung followed by the liver and spleen in the animal model [84]. CIK cells are heterogenous T cells, composed of CD3^+^CD8^+^, CD3^+^CD4^+^, CD3^+^CD56^+^ subpopulations and the main dominant of CIK cells is TCR αβ T cells [14]. Mechanistically, cell signaling not only through TCR/CD3 but also through NKG2D, DNAM-1, and NKp30 leads to CIK cell activation resulting in granule exocytosis, cytokine secretion, and cytotoxicity [14, 85]. FasL is another major effector mechanism used by CIK cells to induce the apoptosis of tumor cells [86]. Alternatively, it has been reported that TCR αβ CD3^+^CD56^+^CIK cells can be retargeted to exert antibody-dependent cellular cytotoxicity (ADCC) function by antigen-specific mAbs [87]. Although CIK/DC-CIK therapy has improved anti-tumor responses in a total of 12 clinical trials targeting lung cancer in recent years [15], the dynamic phenotype profiles of checkpoint molecules on CIK cells derived from patients with NSCLC patients has revealed that CIK cells may partly be exhausted before the clinical transfusion. This has been also characterized by the elevated expression of PD-L1, LAG-3, TIM-3 and the reduced expression of PD-1 and CTLA-4. Based on these findings, blocking PD-1/PD-L1 improve the efficiency of CIK therapy for NSCLC patients [88]. Furthermore, a clinical report on the enhancement of autologous CIK cells after treatment with PD-1 blocking antibodies in patients with advanced NSCLC provided additional evidence for this combination strategy [89].

Alternatively, in a phase I clinical trial in patients with advanced solid tumors including NSCLC, pembrolizumab-activated DC-CIK cells demonstrated superior antitumor properties with increased IFN-γ secretion in ex vivo and an overall disease control rate of 64.5% [90]. A case report of a 63-year-old man with squamous cell carcinoma has provided further clinical evidence. The patient received a diagnosis of squamous cell carcinoma after biopsy of the right lower lobe lung mass and had developed multiple metastases on CT scans. After first-line and second-line chemotherapy, the disease was processed. After receiving pembrolizumab in combination with seven cycles of CIK cell transfer, the patient continued to be in remission 185 days posttreatment and had no adverse events. The tumor cells from pretreatment biopsies showed strongly expression of PD-L1 instead of PD-1, and large number of tumor-infiltrating CD3^+^ T cells were observed [91]. Taken together, this study suggested that a combination of CIK cells and pembrolizumab could synergistically enhance therapeutic efficacy in metastatic NSCLC patients, especially when PD-L1 expression in tumor biopsies was high. Han Y. et al. also documented that autologous CIK cells improved the clinical response to PD-1 blocking antibodies in patients with advanced NSCLC [89]. Recently, a Phase IB Trial of autologous CIK cells in combination with Sintilimab, (mAb PD-1), plus chemotherapy in patients with advanced NSCLC also showed encouraging efficacy (NCT03987867) [92]. Additionally, Mankor JM. et al. observed that the efficacy of nivolumab and ipilimumab in patients with malignant pleural mesothelioma was related to a subtype of cytotoxic T cells effector memory [93]. Therefore, it is important to stratify patients using accessible markers of T-cell status or tumor genomic detection to design therapeutic trials.

### PD-1/PD-L1 blockades in CAR T-cell therapy in lung cancer

The concept of adaptive immunotherapy resurfaced with the advent of CAR T-cell therapy. The first report of a combined approach of CAR T cells with PD-1 blockade in Her-2 transgenic mice was reported in 2013 [94, 95]. It was demonstrated that anti-PD-1 antibody can potently enhance CAR T-cell therapy by the eradication of established tumors without severe toxicity. Beyond PD-1 blockade, constructed CAR T cells that target PD-L1 were found to exert cytotoxic activity against PD-L1^high^ NSCLC cells and xenograft tumors. An additional subtherapeutic dose of local radiotherapy improved the efficacy of PD-L1-CAR T cells against PD-L1^low^ NSCLC cells and tumors [96]. Novel CAR.αPD1-T cells were generated based on the anti-CD19 CAR, and constitutive anti-PD-1 secretion was more efficient in tumor eradication than parental anti-CD19 CAR T cells in human lung carcinoma xenograft tumors [97]. Due to dual expression of mesothelin (MSLN) in both lung cancer and normal mesothelium, the development of mesothelin-specific CAR-T cell therapy that incorporates an HLA-gated safety mechanism can selectively kill MSLN(+)A*02(-) malignant cells [98]. Furthermore, image-guided intrapleural delivery of CAR T cells using intracavitary or intratumoral routes and pembrolizumab treatment after CAR-T infusion is feasible, repeatable and safe across anatomically variable pleural cancers (NCT 02414269) [99].

Currently, there are 17 clinical trials worldwide investigating the safety and efficacy of CAR-T cell therapy in the treatment of lung cancer. They are specifically targeting epidermal growth factor receptor (EGFR); human epidermal growth factor receptor 2 (HER2); mesothelin (MSLN); prostate stem cell antigen (PSCA); mucin 1 (MUC1); carcinoembryonic antigen (CEA); tyrosine kinase-like orphan receptor 1 (ROR1); programmed death ligand 1 (PD-L1) and CD80/CD86. There are three clinical trials involving PD-1/PD-L1 (NCT03525782, NCT02862028 and NCT03198052). In NCT03525782, 8 patients with NSCLC (IIIb to IV stage) were infused with anti-MUC1 CAR-T cells combined with PD-1 knockout engineered T cells. All patients experienced significant symptoms in the first 2 weeks after infusion. Serum CYFRA 21, a tumor marker of squamous cell carcinomas, declined following infusion and subsequently increased 4 weeks after treatment. In 2/6 patients, lung tumor size shrunk significantly within 4 weeks after treatment, while the effect on metastasis was limited. No cytokine release syndrome (CRS) or adverse effects were observed in patients [100]. This study suggests that combined MUC1-CAR^+^/PD-1-KO therapy is feasible but effective individually. Additional studies of CAR-T in LC are ongoing (NCT02862028, NCT03198052).

To date, one phase 1 clinical trial (NCT03392064) has been conducted using AMG 119, a chimeric antigen receptor (CAR) T cell therapy targeting DLL3 for the treatment of relapsed/refractory SCLC patients [101]. The target gene in this study is delta-like ligand 3 (DLL3), an inhibitory ligand of Notch receptors that regulate neuroendocrine differentiation in SCLC. Given the serious side effects of CAR-T cells targeting healthy tissue [102, 103], DLL3 is highly restricted to SCLC with a neglectable expression in the normal adult tissues, making it an ideal target for cancer immunotherapy [104]. A new clinical trial using DLL3-directed chimeric antigen receptor T-cells in patients with extensive stage small cell lung cancer (NCT05680922) has also been listed but not yet recruiting. Another study in in vitro and xenograft mouse models demonstrated that the PD-1 inhibitory antibody dramatically improved the anti-tumor efficacy of the DLL3 bispecific antibody [105], suggesting the feasibility of DLL3 CAR-T combined with PD-1 inhibitors. In addition, regional mesothelin-targeted CAR T-cell therapy in patients with malignant pleural disease, in combination with the anti-PD-1 agent pembrolizumab showed promising outcomes in a Phase I Trial [106]. Alternatively, a phase I clinical trial to evaluate the safety and tolerability of autologous mesothelin (MSLN)-targeted chimeric antigen receptor (MSLN-CAR) T cells secreting PD-1 nanobodies (αPD1-MSLN-CAR T cells) in patients with solid tumors achieved good outcomes (NCT04503980). All patients showed expansion of CAR-T cells and increased PD-1 nanobodies in circulation. The CAR T-cell therapy is a relatively safe therapeutic option safe and the total objective response rate was 63.64% [107].

Recently, a Phase I study of CAR-T cells with PD-1 and TCR disruption in mesothelin-positive solid tumors establish the preliminary feasibility and safety of CRISPR-engineered CAR-T cells with PD-1 disruption (NCT03747965). Lung squamous cell carcinoma cell line NCI-H226 was used in vitro experiment. PD-1/PD-L1 pathway inhibits CAR-T cell function in vitro. knocking out the PDCD1 gene would enhance the antitumor effect of CAR-T cells. PDCD1 knockout P4 (MPK-CAR-T) cells were generated via electroporation of Cas9-sgRNA ribonucleoprotein (RNP), had substantial cytotoxic effects against the cell line PD-L1^+^ cell line and released substantial amounts of IFN-γ in vitro. A total of 15 patients received one or more infusions of MPTK-CAR-T cells without prior lymphodepletion. No dose-limiting toxicity or unexpected adverse events were observed in any of the 15 patients. The best overall response was stable disease (2/15 patients) [108]. Therefore, we conclude that CAR T-cells are uniquely equipped with specific molecules and a combination with PD-1/PD-L1 blockade is feasible. Continued safety monitoring in future trials is essential.

### Blockade of PD-1 immunosuppression enhances γδ T cells therapy in lung cancer

δ T cells represent a unique T cell subpopulation capable of recognizing cancer cells not only through a TCR-dependent pathway, but also through natural killer receptors (NKRs) that are not restricted to the major histocompatibility complex (MHC). As aforementioned, human Vδ1 T cells exerted anti-tumoral function in the tissues of NSCLC patients [57]. Human Vδ1 T cells can be isolated from the peripheral blood mononuclear cells (PBMC) of healthy donors and proliferated in vitro induced by mitogen concanavalin A (Con A) [109]. The cytotoxic Vδ1 T cells against B-cell chronic lymphocytic leukemia cells were correlated with the expression of Vδ1 TCR, CD56, and CD95. In contrast, using the same expansion method, Vδ2 T cells can be expanded by targeting chronic myeloid leukemia-derived cells, which correlate with Vδ2 TCR and NKG2D [109]. Although peripheral blood Vδ1 T cells typically represent a minority population compared to the more dominant Vγ9Vδ2 [110], allogeneic CAR Vδ1 T cells expanded from PBMCs and genetically modified to express a 4-1BB/CD3z CAR against GPC-3 display robust antitumor efficacy against hepatocellular carcinoma [111].

Human Vγ2Vδ2 T cells constitute important circulating γδ T cells that bridge adaptive and innate immunity. γδ T cells can be effectively expanded using synthetic antigens such as pyrophosphomonoesters and nitrogen-containing bisphosphonates (N-BPs). Adoptive transfer of autologous or allogeneic Vγ9Vδ2-T cells expanded with zoledronate in patients with refractory NSCLC [17, 112, 113] resulted in good antitumor responses. High PD-1 levels on the activated γδ T cells also suggest the potential of combination therapy involving γδ T cells and PD-1 ICIs. Of particular interest, HDAC inhibitors attenuate the antitumor cytotoxic potential of γδ T cells, whereas PD-1 blockade enhances the antitumor effector functions of HDAC inhibitor-treated γδ T cells [114]. However, Vδ T cells are double-faced immunocytes in cancer treatment. The interaction between PD-1 on αβ T cells and its ligand PD-L1 on γδ T cells restrains αβ T cell activation [115]. It is reasonable to conduct γδ T cell functional identification and elimination of suppressive subgroup before transferring the γδ-T cells into the patients. In addition, CD16 has been shown to mediate ADCC in γδ T cells by PD-1 blockade in follicular lymphoma [116]. Considering the percentage of γδ CD16^+^CD3^+^CD56^+^ CIK cells accounts for approximately 31% of total CD16^+^ CIK cells [87], isolation and expansion of this subset of CIK cells may expand the scope of immune therapy in the future.

Three clinical trials using γδ-T cells against lung cancer have been reported. NCT03183232 was originally designed to evaluate the safety and efficiency of autologous PBMC-derived γδ-T cells against lung cancer. However, PBMCs from the majority of cancer patients cannot be effectively expanded in terms of cell number, purity and function. Moreover, cancer patients cannot afford to donate 100 ml of blood for culture every 2–3 weeks. Therefore, they optimized the protocol of ex vivo expansion of Vγ9Vδ2-T cells derived from allogeneic PBMCs. The clinical trial has been completed but the results are not yet available. Another clinical trial NCT02459067, designed to determine the safety, tolerability, maximum tolerated dose (MTD) and efficacy of Autologous γδ T Lymphocytes (ImmuniCell®) in patients with melanoma, renal cell cancer (RCC) or non-small cell lung cancer (NSCLC) has been terminated. Taken together, γδ T-cell therapy for lung cancer is still in its early stages, and clinical data is inadequate. An overview of three types of adaptive immunotherapies in clinical trials involving the PD-1/PD-L1 axis is shown in Table 1.

**Table 1:** Completed and ongoing clinical trials

Study/drug	Target population	Study design	Treatment	Key inclusion criteria	Key results summary
CIK					
NCT03987867 [92] Autologous CIK cells with PD-1 inhibitor (IBI308, sintilimab) and chemotherapy	Advanced NSCLC	n = 30 Objective response rate (ORR) was calculated by the percentage of patients with a confirmed complete (CR) or partial response (PR).	IBI308 intravenous infusion 200 mg d1; Pemetrexed intravenous infusion 500 mg/m² d2 or Liposome paclitaxel intravenous infusion 135 mg/m² d2; Carboplatin intravenous infusion AUC5 d2; CIK cells, 1 × 10^10^ (10 billion), intravenous infusion, d14; Q3W.	Age 18–75 years; Patients with stage IIIB/IIIC/IV NSCLC; Adenocarcinoma with wild type of EGFR gene and ALK fusion gene negative can be included;	The ORR were 82.4%; Median PFS was 19.3 months; autologous CIK cells immunotherapy in combination with sintilimab plus chemotherapy was well tolerable and showed encouraging efficacy in patients with previously untreated, advanced NSCLC.
NCT04836728 Autologous CIK cells with PD-1 inhibitor (IBI308,sintilimab) and chemotherapy A Randomized, Multicenter, Open-label Phase II Study	Stage IV NSCLC	n = 156 A Randomized, Multicenter, Open-label Phase II Study Overall survival Progression-free survival	IBI308 intravenous infusion 200 mg d1; Pemetrexed intravenous infusion 500 mg/m² d2 or Albumin paclitaxel intravenous infusion 260 mg/m² d2; Carboplatin intravenous infusion AUC5 d2; CIK cells, 1 × 10^10^, intravenous infusion, d14; Q3W.	Age 18–75 years; stage IV NSCLC; adenocarcinoma with wild type of EGFR gene and ALK fusion gene negative can be included.	Ongoing
**CAR-T**					
NCT02414269 [99] Autologous T cells genetically engineered to target the cancer-cell surface antigen mesothelin with pembrolizumab	Malignant pleural mesothelioma; non-small cell lung cancer metastatic to the pleura; breast cancer metastatic to the pleura	n = 113 Composite measure of severity and number of adverse events (AEs); response of complete response (CR); partial response (PR); and stable disease (SD);serum levels of the biomarker soluble mesothelin related peptide (SMRP) after treatment	cyclophosphamide intravenously (at 1.5 g/m²), 2–7 days before T cell infusion; Administration through the pleural catheter- On day 0 patients will be treated with genetically modified T cells. Pembrolizumab 4 weeks (+ 3/-1 week window) after completing CAR T cell administration.	Age ≥ 18 years; Karnofsky performance status ≥ 70%; Malignant pleural mesothelioma; non-small cell lung cancer or breast cancer metastatic to the pleura; Mesothelin expression (> 10% of the tumor expressing mesothelin) by IHC analysis; Elevated serum SMRP levels (> 1.0 nM/L).	CAR T cells were detected in peripheral blood for > 100 days in 39% of patients. Median overall survival from CAR T-cell infusion was 23.9 months (1-year overall survival, 83%). Stable disease was sustained for ≥ 6 months in 8 patients; 2 exhibited complete metabolic response on PET scan.
NCT03525782 [100] Anti-MUC1 CAR T Cells and PD-1 Knockout Engineered T Cells	Advanced NSCLC	n = 8 Following treatment, levels of lymphocytes, IL-6, hs-CRP, PCT, CYFRA21, NSE(E), and SCC were monitored at regular intervals. Changes in tumor size were examined by MRI scans.	MUC1-specific CARs were constructed using the SM3 scFv. PD-1 gene KO in the CAR positive T cells was achieved using the CRISPR-Cas9 system and validated by sequencing. MUC1-CAR+/PD-1- KO engineered T cells at a dose of 2.5 × 10²/KG were infused over 60 min.	Age 36 to 84 years; MUC1 is expressed in malignancy tissues by immuno-histochemical (IHC); ECOG performance status of 0–1 or karnofsky performance status (KPS) score is higher than 60; Patients have a life expectancy > 12 weeks.	All patients had significant symptom improvements in the first 2 weeks after infusion. Serum CYFRA 21 declined following infusion and subsequently increased 4 weeks after treatment. In 2/6 patients, lung tumor size shrunk significantly within 4 weeks. No other adverse effects.
NCT03198052 CAR-T Cells Targeting PSCA, MUC1, TGFβ, HER2, Mesothelin, Lewis-Y, GPC3, AXL, EGFR, B7-H3 or Claudin18.2	Lung Cancer	n = 30 Number of Patients with Dose Limiting Toxicity; Percent of Patients with best response as either complete remission or partial remission; Median CAR-T cell persistence.	3 or more cycles of the CAR-T cells treatment via systemic or regional injection, from 1 × 10^6^/kg-10 × 10^6^/kg weight.	Advanced cancer that expresses PSCA, MUC1, GPC3, AXL, EGFR or B7-H3 protein; Autologous transduced T cells with greater than or equal to 20% expression of PSCA, MUC1, GPC3, AXL, EGFR or B7-H3 CAR determined by flow cytometry and killing of PSCA, MUC1, GPC3, AXL, EGFR, or B7-H3-positive targets greater than or equal to 20% in cytotoxicity assay.	Recruiting
NCT04503980 αPD1-MSLN-CAR T Cells [107]	MSLN-positive advanced solid tumors	n = 10 Maximum tolerated dose (MTD); Objective response rate (ORR); Progression-free survival (PFS); Overall survival (OS); Peak Plasma Concentration (Cmax); Pharmacodynamics (PD).	Four doses of CAR T cells will be evaluated in this study: 1 × 10^5^ CAR^+^ T cells/kg, 3 × 10^5^ CAR^+^ T cells/kg, 1 × 10^6^ CAR^+^ T cells/kg, 3 × 10^6^ CAR^+^ T cells/kg.	Age 18-70 years; advanced solid tumors; ECOG performance status of 0 or 1; Staining of MSLN must be greater than 50%; of the cells in the tumor tissue, apparent expressing in the membrane; PD-L1 expression must be positive.	The total objective response rate was 63.64%. All enrolled patients are still alive. The longest PFS was up to 26 months. Median follow-up was four months.

ECOG performance: Eastern Cooperative Oncology Group performance; IHC: immunohistochemistry; CRS: cytokine release syndrome

## Conclusion and future perspectives

A few clinical trials of adaptive T-cell therapy with PD-1/PD-L1 blockade have been conducted in recent years. However, understanding the impact of the PD-1/PD-L1 axis on lung cancer is still in the nascent stages. More importantly, we have paid little attention to PD-L2 (a second ligand of PD-1), despite the fact that its expression has been upregulated in patients with lung adenocarcinoma [117, 118] and has the potential to serve as a clinicopathological and prognostic marker [119]. Undeniably, PD-L2 has previously been considered an insignificant ligand, although it binds to PD-1 with a 2-6-fold higher affinity [120]. It can inhibit T cell activation via recruiting SHP-2, yet it does not inhibit T cells due to cell cycle arrest in G0/G1. This characteristic is similar to that of CTLA-4. Notably, cemiplimab, a human PD-1 monoclonal antibody that binds to PD-1 and blocks its interaction with PD-L1 and PD-L2 has been approved by FDA in 2018 [121]. In fact, cemiplimab monotherapy significantly improved overall survival and progression-free survival compared to chemotherapy in patients with advanced NSCLC with PD-L1 of at least 50% [122, 123], providing another option for immune therapy.

Secondly, the moderate understanding of the tumor microenvironment is still a major hurdle to the complete success of lung cancer immunotherapy. For instance, lower airway dysbiosis led to the activation of checkpoint inhibitor [124]. Of importance, this lower airway dysbiotic signature was found to be more prevalent in the stage IIIB-IV tumor-node-metastasis lung cancer group, associated with poor prognosis and accompanied by the upregulation of the IL-17, PI3K, MAPK, and ERK pathways in airway transcriptome. It is also important to mention about microbiome, it has been suggested that normal human oral flora, *Veillonella parvula*, may act as a key driver force in lung cancer [125]. Moreover, use of antibiotics during ICIs treatment regimen has been shown to significantly decrease their efficiency against lung cancer [126, 127]. In addition, the relationship of lung microbiota to PD-1/PD-L1 inhibitors responses has been investigated [128]. CXCL9 levels in bronchoalveolar lavage fluid (BALF) were significantly elevated in responders compared with non-responders, along with a greater diversity of the lung microbiome profile in BALF and a greater frequency of the CD56^+^ subset in blood T cells before initiating nivolumab. Antibiotic treatment in a preclinical lung cancer model significantly decreased CXCL9 in the lung TME, resulting in reduced sensitivity to anti-PD-1 antibody. Unfortunately, authors could not identify the specific bacterial species involved in the therapeutic effect of ICIs in this study [128]. Further research is needed to fully understand how lung microbiota can more specifically influence the outcome of cancer immunotherapy. Interestingly, Ochi N. et al. suggested that antibiotics treatment was significantly associated with a shorter median overall survival (OS), but not progression-free survival (PFS) in NSCLC patients who received antibiotics before or after ICI treatment in a retrospective study. The detrimental effect of impact of antibiotics uses on the efficacy of ICIs differed based on PD-L1 expression in patients with advanced NSCLC [129]. However, this effect was not observed at the multivariate analysis. The influence factor on overall survival needs to be explored further.

Thirdly, PD-1/PD-L1 inhibitors might promote the efficacy of CAR-T/CAR-CIK, bispecific antibody or δ T cells, however, this assumption requires experimental validation. Notably, a recent clinical trial NCT02425748 was designed to evaluate the safety and efficiency of γδ T cells in combination with DC-CIK against NSCLC (without EGFR mutation) compared to either monotherapy of DC-CIK or γδ T cells. Although the results have not been published, this study offers another promising immunotherapy approach.

Finally, a very limited number of reports are available on clinical trials of adaptive immunotherapy in SCLC. For instance, there was only one ongoing clinical trial of SCLC registered as NCT02688673. This study aims to evaluate the safety and efficacy of recombinant adenovirus-code MUC1 and Survivin-transfected dendritic cells (DC) combined with CIK cell treatment in patients with extensive-stage SCLC. Furthermore, a combination of bispecific DLL3-targeted antibody and PD-1 inhibition retained the growth of SCLC more efficiently [105]. Thus, it may also be potentially envisioned as a novel strategy for SCLC.

Similarly, recent observation suggested that PD-L1/PD-1 blockage enhanced the cytotoxicity of natural killer (NK) cells on NSCLC by granzyme B secretion. This finding was also demonstrated in NK cells isolated from NSCLC patients. Their outcomes highlight that PD-L1/PD-1 blockade enhances cellular adaptive immune therapy [130].

In this review, we emphasize the role of three T cell adoptive therapeutic approaches and discuss whether PD-1/PD-L1 blockade can effectively ‘unleash’ T cell response. In our opinion, PD-1/PD-L1 blockade could give a boost to T-cell immunotherapy if patient-centered trials can be considered.

## Data Availability

Not applicable.

## Abbreviations

CIK cells: cytokine-induced killer cells
CAR-T cells: chimeric antigen receptor T-cells
PD-1: programmed cell death protein 1
PD-L1: programmed death ligand 1
NSCLC: non-small cell lung cancer
SCLC: small cell lung cancer
ICI: immune checkpoint inhibitors
CSC: cancer stem cell
TAMs: tumor-associated macrophages
CAF: cancer-associated fibroblasts
